# Cilostazol Combats Lipopolysaccharide-Induced Hippocampal Injury in Rats: Role of AKT/GSK3*β*/CREB Curbing Neuroinflammation

**DOI:** 10.1155/2024/3465757

**Published:** 2024-09-26

**Authors:** Doaa Abou El-ezz, Waleed Aldahmash, Tuba Esatbeyoglu, Sherif M. Afifi, Marawan Abd Elbaset

**Affiliations:** ^1^ Pharmacology and Toxicology Department Faculty of Pharmacy October University for Modern Sciences and Arts University, Giza 12556, Egypt; ^2^ Department of Zoology College of Science King Saud University, P.O. Box 2455, Riyadh 11451, Saudi Arabia; ^3^ Department of Molecular Food Chemistry and Food Development Institute of Food and One Health Gottfried Wilhelm Leibniz University Hannover, Am Kleinen Felde 30, Hannover 30167, Germany; ^4^ Department for Life Quality Studies Rimini Campus University of Bologna, Corso d'Augusto 237, Rimini 47921, Italy; ^5^ Department of Pharmacology Medical Research and Clinical Studies Institute National Research Centre, Dokki, Giza 12622, Egypt

## Abstract

Neuroinflammation is important in the pathophysiology of several degenerative brain disorders. This study looked at the potential neuroprotective benefits of cilostazol, a phosphodiesterase inhibitor, against LPS-induced hippocampus damage in rodents and the principal molecular involvement of AKT/GSK3*β*/CREB signaling pathways. Behavioral tests revealed that cilostazol successfully corrected LPS-induced neurobehavioral impairments. Furthermore, cilostazol therapy lowered hippocampal levels of amyloid beta 1–42 (A*β*1-42) and p-tau protein, both of which are critical pathological indicators of neurodegenerative disorders. Furthermore, cilostazol administration suppressed LPS-induced rises in hippocampus caspase-3 and NF-*κ*B levels while elevating rat B-cell/lymphoma 2 (BCL2) and brain-derived neurotrophic factor (BDNF) levels, which are implicated in neuronal survival and synaptic plasticity. Cilostazol treatment also restored the decreased phosphorylation of protein kinase B (p-AKT) and reduced the elevated levels of phosphorylated glycogen synthase kinase-3 beta (p-GSK3*β*) and cAMP response element-binding protein (CREB) in the hippocampus of LPS-treated rats. Histopathological examination revealed that cilostazol ameliorated LPS-induced brain damage with reduced neuronal loss and gliosis. Immunohistochemistry analysis showed a decrease in Iba-1 expression, indicating a reduction in microglial activation in the cilostazol-treated group compared to the LPS group. The findings advocate that cilostazol exerts neuroprotective effects against LPS-induced hippocampal injury by modulating the AKT/GSK3*β*/CREB pathway and curbing neuroinflammation. Cilostazol may hold promise as a therapeutic agent for neuroinflammatory conditions associated with neurodegenerative diseases.

## 1. Introduction

Neuroinflammation involves brain and spinal cord inflammation. Inflammation is a complicated immunological response to infections or tissue injury involving the liberation of cytokines, chemokines, and reactive oxygen species [1]. Infections, traumatic brain damage, stroke, and neurodegenerative illnesses including multiple sclerosis, Parkinson's, and Alzheimer's, and autoimmune disorders such as lupus and rheumatoid arthritis may cause neuroinflammation. Chronic neuroinflammation may worsen these disorders [2, 3]. Neuroinflammation can have both positive and negative effects on the central nervous system (CNS). In the short term, inflammation may help eliminate infections and promote tissue repair. However, persistent inflammation can lead to tissue damage, loss of neurons, and the progression of neurodegenerative conditions. In addition, cognitive impairment and emotional disturbances may arise as consequences of neuroinflammation [4]. Neuroinflammation is linked to neuron inflammatory reactions. Intravenous lipopolysaccharide (LPS), an endotoxin from negative Gram bacteria, causes this neuroinflammation. This method may cause AKT survival pathway suppression, tau hyperphosphorylation, beta-amyloid plaque buildup, and programmed cell death [5]. LPS has been used extensively in animals to investigate the molecular basis of cognitive impairment induced by neurons' inflammatory responses and to develop a targeted treatment for neurological symptoms [6].

It is worth mentioning that neuroinflammation may require treating the underlying illness and employing anti-inflammatory drugs to lessen the inflammation and its impact on the CNS [7, 8].

Cilostazol, an FDA-approved phosphodiesterase III inhibitor, prevents platelet aggregation and has antithrombotic and vasodilatory effects in cerebral ischemia [9]. A randomized, double-blind, multicenter clinical study found that cilostazol helped cerebral infarction patients [10]. Previous research showed that neuronal cilostazol protects against 3-NP-induced HD by changing the Akt/GSK-3/CREB pathway [11]. Based on this background, the current investigation was conducted to investigate the impact of cilostazol on the neuropathological and behavioral derangements generated by LPS in rats. Furthermore, it expanded to investigate potential integrative signals that might provide neuroprotection against the LPS model.

## 2. Materials and Methods

### 2.1. Animals and Experimental Design

Adult male Wistar Albino rats were purchased from the Modern Veterinary Office in Giza, Egypt. All rats weighed between 100 and 150 grams. They were kept at the animal house in the October University for Modern Sciences and Arts (MSA University, Giza, Egypt) in cages that were spacious enough, with a reasonable number of rats in each cage. The surrounding temperature was kept at 24-25°C, and the room was always well-ventilated with 12 h of sunlight and 12 h of dark per day. The rats were provided fresh tap water daily and food in the sort of food pellets. All animals were treated according to the recommendations from the “National Institutes of Health (NIH) Guide for Care and Use of Laboratory Animals” and the study conformed with the ARRIVE guidelines. The experimental protocol was approved by the Ethical Committee at the MSA University, ethics form no. PH6/REC6/2023PhD. Animals' suffering was brought to a minimum whenever possible.

**Figure 1:**
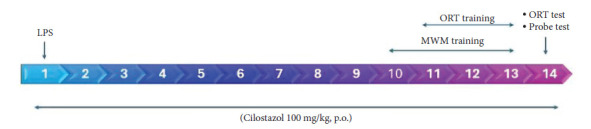
Detailed experimental timeline.

Thirty rats (8–10 weeks old) were randomly assigned into three groups, ten rats in each group. A simple randomization method using a table of random numbers generated from the GraphPad website for random assignment was utilized to assign the rats to the different treatment groups (control, LPS, and LPS + cilostazol) in an unbiased manner. In addition, the researchers were blinded to the group assignments during all experiment phases, including behavioral testing, sample collection, and data analysis. All rats in the first group were given intraperitoneal (IP) phosphate buffer saline (PBS) and considered as a negative control set. The animals in the second group were IP injected with LPS (1 mg/kg) dissolved in PBS to induce neuroinflammation on day 1 of the experiment [12], while the rats in the third group received LPS (1 mg/kg) on day 1 in addition to orally given cilostazol 100 mg/kg daily for two weeks, following the induction with LPS [11] (Figure 1).

### 2.2. Drugs and Chemicals

Lipopolysaccharide (LPS) serotype (055: B5) (Sigma-Aldrich, MO, USA) was dissolved in saline and injected intraperitoneally (IP). Otsuka Pharmaceuticals (UK) provided cilostazol, which was administered orally (PO).

### 2.3. Behavioral Study

#### 2.3.1. Object Recognition Test (ORT)

For three days before testing, each rat had two minutes to explore the apparatus in the dark. Two 2-minute trials (T1 and T2) were performed on testing day. The trial (T1) placed two identical items at opposing corners of the device. The rat discovered these two similar things in the apparatus. After T1, the rat returned to its cage for a 1 h intertrial break. T2 was the “choice” trial when a new object substituted one from T1. Rats again saw the familiar (F) and the new (N) object. After each trial, the apparatus and items were meticulously cleaned with an alcohol solution to avoid a scent effect. The object was found by pointing the nose at it or touching it. Rats' exploration timings were recorded in T1 and T2.

The discrimination index (DI), calculated as a percentage of the total time spent examining the two items in T2, shows the difference in exploration time *N* − *F*/*N* + *F*; DI [13].

#### 2.3.2. Morris Water Maze (MWM) Test

MWM testing was performed in a 180 cm diameter pool with a 70 cm deep output faucet. White, nontoxic, water-based stain paint filled the pool, and warm water kept it at 25 ± 0.5°C. A 9 cm diameter escape platform was in the center of one of the pool's four imaginary quadrants. Before the acquisition or probing test, the rats swam freely for one minute.

Rats were trained for four days, with three sessions of 60 seconds per day. In each session, rats were allowed to freely access the target quadrant platform. After platform repositioning, the animal may stay for 10 s at least. Animals were gently placed on the platform for 20 seconds if they did not reach there in 60 seconds. Spatial learning was measured by the escape latency, the mean of all daily acquisition stage trials, to determine the platform. On the fifth day of the probe test, the platform was demolished, and each rat was placed into the water from the quadrant across from the platform quadrant, with their backs to the pool wall, and given 60 seconds to explore the water. The percent quadrant time was the time spent swimming in the target quadrant and looking for the platform [14].

### 2.4. Histological Study

The rats were euthanized by exsanguination after the behavioral tests, and the brains were quickly dissected, completely cleaned with ice-cold PBS, and blotted dry. The brains were extracted and fixed in 10% neutral buffered formalin for 24 hours for histological and immunohistochemical analysis. The fixed brain tissues were dehydrated in graded ethanol solutions, cleared in xylene, and embedded in paraffin wax. Coronal sections of 5 *μ*m thickness were cut using a rotary microtome and mounted on glass slides [15].

For H & E staining, the sections were deparaffinized, rehydrated, stained with Harris hematoxylin for 5 minutes, differentiated in 1% acid alcohol, blued in ammonia water, counterstained with eosin Y for 2 minutes, dehydrated, cleared, and mounted with DPX mountant.

For Iba-1 immunohistochemistry, the sections were deparaffinized, rehydrated, and subjected to antigen retrieval by heating in 10 mM sodium citrate buffer (pH 6.0). Endogenous peroxidase activity was quenched using 3% hydrogen peroxide. After blocking with 5% normal goat serum, the sections were incubated overnight at 4°C with rabbit anti-Iba-1 primary antibody (1 : 500 dilution, Wako Pure Chemical Industries, Japan). Sections were then incubated with biotinylated goat anti-rabbit IgG secondary antibody, followed by avidin-biotin complex (ABC) reagent (Vector Laboratories, USA). Color was developed using a 3,3′-diaminobenzidine (DAB) substrate, and sections were counterstained with hematoxylin.

The H & E and Iba-1-stained sections were examined under a light microscope. To quantify Iba-1 expression, five random fields per section were analyzed using ImageJ software (NIH, USA) to determine the percentage area of positive staining.

### 2.5. Biochemical Study

#### 2.5.1. Preparation of Brain Tissue Homogenate

The latter weighted portion of each hippocampus tissue was homogenized with ice-cold saline (Medical Instruments, MPW-120, Warsaw, Poland) to produce a 20% w/v homogenate. To remove cell debris, the homogenate was spun for 10 min at 4°C in a cooling centrifuge (Laborzentrifugen, 2k 15, Sigma, Osterode am Harz, Germany). At −80°C, the aliquot was frozen for biological investigation [16].

#### 2.5.2. Determination of Tissue Protein

According to the instructions, the tissue protein content was calculated (cat#: 2624800021730) using the protein estimate kit (Bangalore Genei, Bangalore, India).

#### 2.5.3. Determination of Hippocampal Biochemical Parameters

Hippocampal biochemical contents were determined with the aid of rat amyloid beta peptide 1–42 (A*β*1-42), rat phospho-tau protein (p-tau) ELISA, rat nuclear factor kappa B (NF-*κ*B), rat brain-derived neurotrophic factor (BDNF), rat cyclic AMP response element-binding protein (CREB), rat phospho cyclic AMP response element-binding protein (p-CREB), rat caspase-3, rat B-cell leukemia/lymphoma 2 (BCL2), rat phospho-glycogen synthase kinase-3 beta, and p-Akt (Phospho-Ser473) kits, obtained from MyBioSource, San Diego, USA (cat#: MBS726579, MBS764464, MBS453975, MBS355345, MBS266581, MBS7255484, MBS018987, MBS452319, MBS730623, and MBS9511022, respectively).

### 2.6. Statistical Analysis

The current data are presented as the mean ± SEM. Except for immunohistochemical scoring, data were processed using one-way ANOVA followed by the Tukey post hoc test; statistical analysis was performed using the nonparametric Kruskal–Wallis *H*-test followed by Dunn's test. *p* < 0.05 was regarded as statistically significant. The GraphPad Prism software (version 9; GraphPad Software Inc., San Diego, CA, USA) was used to execute the statistical analysis and create the graphical representation.

## 3. Results

### 3.1. Assessment of Cilostazol Effect on Behavioral Tests in LPS-Induced Neurobehavioral Deficit

**Figure 2:**
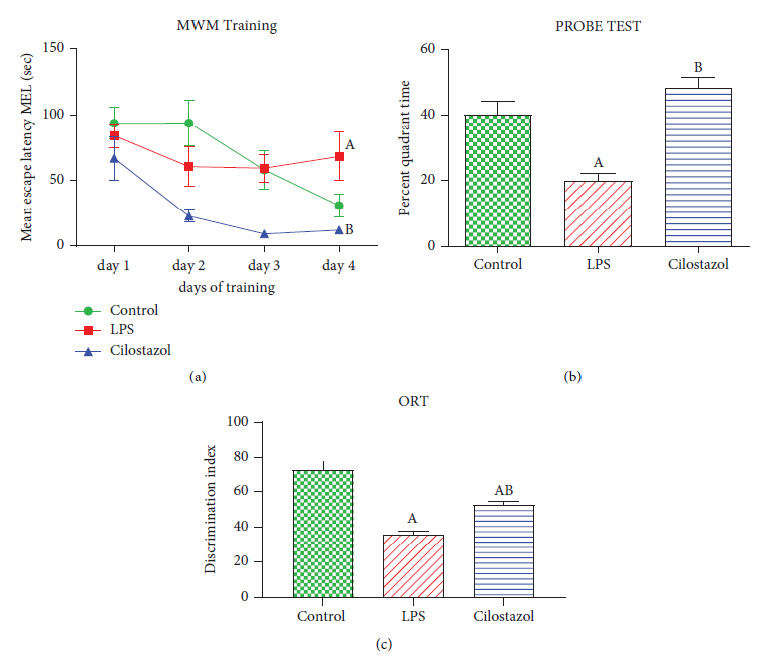
Effect of cilostazol on spatial memory (MWM) test and recognition memory (ORT) in LPS-induced neurobehavioral deficit (*n* = 10). Mean escape latency (MEL): (a) probe test; (b) discrimination index in ORT; (c) bars are presented as mean ± SEM. A *P* < 0.05 vs. control; B *P* < 0.05 vs. LPS.

On day 4, the LPS-treated rats and the control group experienced a substantial increase in training time delay. In the probe study, rats given cilostazol spent much more time in the target quadrant than in the LPS group. These findings were statistically indistinguishable from those obtained by the normal control group (Figures 2(a) and 2(b)).

In a similar manner, the ORT discrimination index was significantly lower in LPS-treated rats than in the control group. The cilostazol group reversed this decrease compared to the LPS group. These findings were statistically distinguishable from those obtained by the normal control group (Figure 2(c)).

### 3.2. Assessment of Cilostazol Effect on Hippocampal A*β*1-42 and p-tau Contents in LPS-Induced Neurobehavioral Deficit

**Figure 3:**
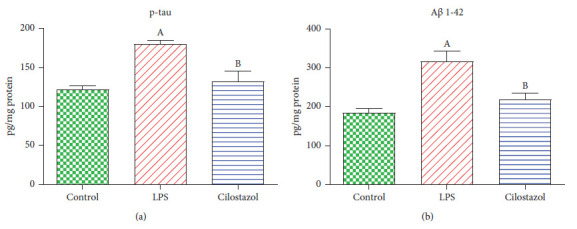
Effect of cilostazol on rat hippocampal contents of phosphorylated tau protein (p-tau) (a) and amyloid beta protein 1–42 (A*β* 1–42) (b) in LPS-induced neurobehavioral deficit (*n* = 10). Bars are presented as mean ± SEM. A *P* < 0.05 vs. control; B *P* < 0.05 vs. LPS.

Lipopolysaccharides significantly elevated A*β*1-42 and p-tau hippocampal contents by 71 and 47% compared to the normal group, respectively, whereas rats receiving LPS + cilostazol exhibited a prominent reduction in the hippocampal contents of A*β*1-42 and p-tau by 31 and 27%, respectively (Figure 3).

### 3.3. Assessment of Cilostazol Effect on Hippocampal Caspase-3, NF-*κ*B, BCL2, and BDNF Contents in LPS-Induced Neurobehavioral Deficit

**Figure 4:**
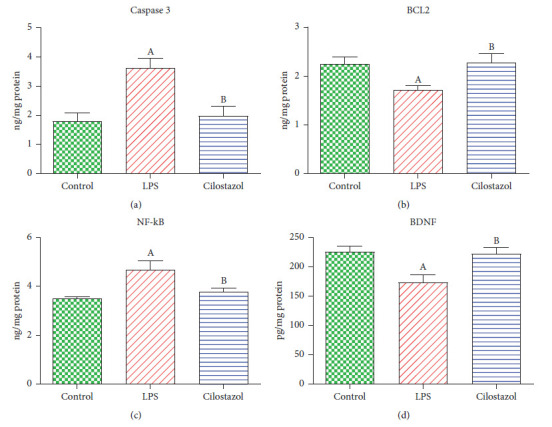
Effect of cilostazol on rat hippocampal contents of caspase-3 (a), B-cell lymphoma 2 (BCL2) (b), nuclear factor kappa B (NF-*κ*B) (c), and brain-derived neurotrophic factor (BDNF) (d) in LPS-induced neurobehavioral deficit (*n* = 10). Bars are presented as mean ± SEM. A *P* < 0.05 vs. control; B *P* < 0.05 vs. LPS.

The LPS-treated group showed increased hippocampal content of caspase-3 and NF-*κ*B by 33% and 98%, against the normal control group, respectively. On the contrary, the administration of cilostazol declined the hippocampal content of caspase-3 and NF-*κ*B by 19 and 46% against the LPS group, correspondingly (Figure 3). Meanwhile, the hippocampal content of BCL2 and BDNF was decreased by about 24% in the LPS-administered rats compared to the normal control group, respectively. On the other hand, the rats given cilostazol revealed higher hippocampal content of BCL2 and BDNF by 33 and 28% than the LPS group, respectively (Figure 4).

### 3.4. Assessment of Cilostazol Effect on Hippocampal p-Akt, p-GSK-3*β*, CREB, and p-CREB Contents in LPS-Induced Neurobehavioral Deficit

**Figure 5:**
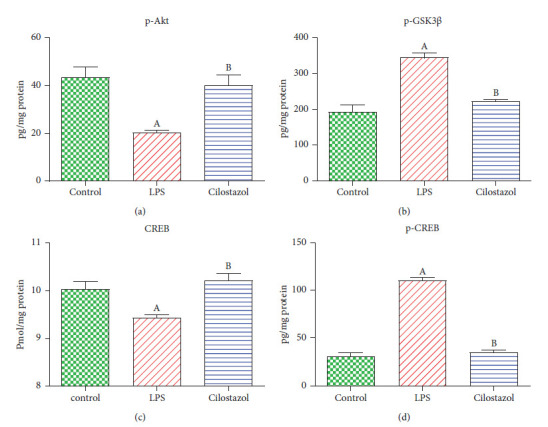
Effect of cilostazol on rat hippocampal contents of phosphorylated protein kinase B (p-Akt) (a), glycogen synthase kinase-3 beta (p-GSK-3*β*) (b), cAMP response element-binding protein (CREB) (c), and phosphorylated cAMP response element-binding protein (p-CREB) (d) in LPS-induced neurobehavioral deficit (*n* = 10). Bars are presented as mean ± SEM. A *P* < 0.05 vs. control; B *P* < 0.05 vs. LPS.

The hippocampal p-Akt content of the LPS group was reduced by 58% compared to the normal control group. Similarly, LPS-treated rats showed a partial decline in the CREB content compared to the normal group. Contrarily, cilostazol markedly restored the hippocampal p-Akt by 97%, compared to the normal control group rats. Besides, the LPS-treated rats boosted the hippocampal p-GSK-3*β* and p-CREB contents by 1.8 and 3.5-fold, compared to the normal rats, respectively. Remarkably, cilostazol decreased hippocampal p-GSK-3*β* and p-CREB by 34% and 69%, matched to the LPS-administered, respectively (Figure 5).

### 3.5. Histopathology

**Figure 6:**
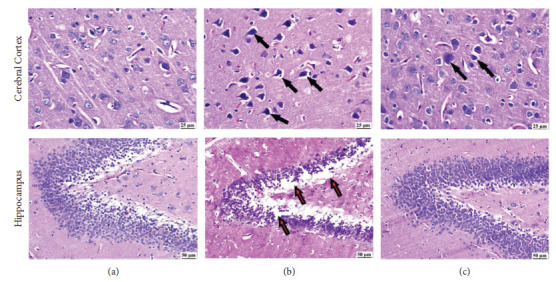
Photomicrograph of the brain (H & E) showing (a) the control group showing normal brain structure, (b) the LPS group showing numerous dark neurons in the cerebral cortex (black arrows) and neuronal loss (red arrow) with vacuolation in the hippocampus, and (c) the cilostazol group showing fewer dark neurons (black arrow) and apparently normal hippocampus.

The LPS-administered group exhibited diffuse gliosis in the cerebral cortex with the existence of numerous dark degenerating neurons as well as neuronophagia. The hippocampus showed neuronal loss with vacuolation. The cilostazol group alleviated brain damage, as only a few dark, shrunken neurons were detected in the cerebral cortex with the apparently normal hippocampus. No histopathological changes were observed in the normal control set (Figure 6).

### 3.6. Assessment of Cilostazol Effect on Iba-1 Expression by Immunohistochemistry

**Figure 7:**
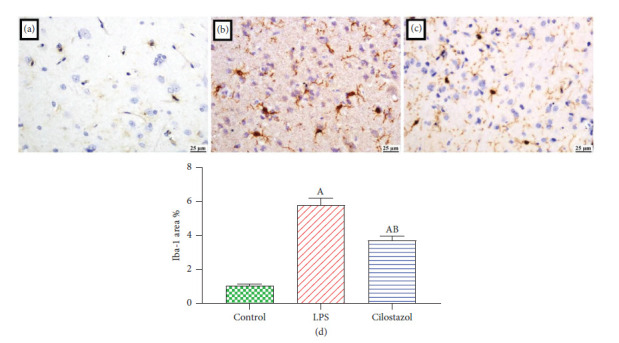
Photomicrographs of the brain (immune staining) showing Iba-1 expression in different experimental groups: (a) the control group showing limited expression, (b) the LPS group showing intense Iba-1 expression, (c) cilostazol showing reduced Iba-1 expression, and (d) chart represents Iba-1 expression in brain sections as area percent. Data are presented as mean ± SEM. A *P* < 0.05 vs. control; B *P* < 0.05 vs. LPS.

A significantly higher level of Iba-1 positive expression was detected in the LPS group. Meanwhile, a significant reduction was observed in the cilostazol-treated group compared to the LPS group (Figure 7).

## 4. Discussion

Neuroinflammation is a common characteristic of neurodegenerative disorders such as Parkinson's disease, multiple sclerosis, and Alzheimer's disease (AD) [17]. In AD, the formation of *β*-amyloid (A*β*) plaques activates an inflammatory pathway that leads to neurotoxicity and neuronal cell death [18]. The causes of these disorders are poorly known, and no effective treatments are presently available [17]. To address this issue, the current research was performed to determine how the medication cilostazol impacts neuropathological and behavioral changes generated by lipopolysaccharides (LPSs) in rats, as well as to identify possible signaling pathways for neuroprotection. Caspase-3, NF-*κ*B, BCL2, and BDNF, which are linked to the development of neurodegenerative illnesses, were shown to be released by microglia upon stimulation with LPS, according to the studies [14, 19–22]. In particular, higher amounts of amyloid *β* 1–42 and p-tau were detected in the hippocampus, a region critical for memory formation and especially susceptible to AD. Amyloid *β*1-42 accumulation contributes to cognitive decline, while aberrant tau phosphorylation affects neuronal function and causes cognitive impairment [23, 24].

It has been shown that cilostazol inhibits macrophage activation and inflammatory responses, as indicated by a decrease in Iba-1 expression, a hallmark of microglia activation. In addition, cilostazol decreased the levels of caspase-3, NF-*κ*B, BCL2, and BDNF, which are related to neuronal death and damage. In addition, cilostazol enhanced cognitive function, as shown by an increase in probe time in the Morris water maze (MWM) test and an increase in the discrimination index in the object recognition test (ORT). The improvement may be attributable to the decreased levels of *β*-amyloid 1–42 and p-tau proteins, which are directly associated with cognitive impairment in AD [25].

The p-AKT/p-GSK-3*β*/p-CREB pathway is involved in the neuroprotective effects of cilostazol. Protein kinase AKT controls cell survival and proliferation [26]. Under typical circumstances, p-GSK-3*β* predominates in response to increased p-AKT. Some studies demonstrated a reduction in p-AKT levels in neuroinflammation, while others demonstrated an increase [11, 27] and others a rise [28, 29]. CREB regulates the p-GSK-3*β* kinase, which is involved in several cellular activities. Various extracellular inputs activate CREB, a transcription factor that controls gene expression in neurons [26, 30]. In earlier research, CREB deactivation modulated BDNF and lowered aberrant tau formation, showing neuroprotective benefits and increased neuronal survival [31]. This is the inaugural study that explores the impact of cilostazol on LPS-induced neuroinflammation and cognitive deficits *in vivo*. We present fresh evidence demonstrating that cilostazol influences the AKT/GSK3*β*/CREB pathway in this model. Our research unveils novel findings about cilostazol's capacity to decrease A*β*1-42 and p-tau accumulation in the hippocampus. By integrating behavioral, biochemical, and histological analyses, we offer a comprehensive perspective on cilostazol's neuroprotective effects.

### 4.1. Limitations

While the present study demonstrates promising neuroprotective effects of cilostazol against LPS-induced neurobehavioral deficits and neuroinflammation, there are some limitations that should be acknowledged which are listed as follows:

1. Dose-response assessments: The current study examined only a single dose of 100 mg/kg cilostazol. Evaluating different dose levels would help determine the minimal effective dose and establish a therapeutic window for cilostazol's neuroprotective effects.
2. Long-term effects: This study focused on short-term treatment over 2 weeks. Investigating the effects of longer cilostazol treatment duration, possibly in a chronic neurodegeneration model, would provide insight into whether the benefits are sustained over time.
3. Additional mechanisms: While the study implicates AKT/GSK3*β*/CREB signaling, a more comprehensive analysis of cilostazol's effects on other inflammatory pathways and neurodegenerative processes could further elucidate its mechanisms of action.
4. Other neuroinflammatory/neurodegenerative models: Evaluating cilostazol in different animal models representing various neurological conditions would demonstrate the breadth of its potential therapeutic applications.
5. The use of single animal species: One potential limitation is the use of a single animal model and species. The experiments were conducted solely in male Wistar albino rats. While rodent models are widely used for preclinical research, there can be limitations in translating findings from a single species to humans. Different species may exhibit varying sensitivity or response to LPS-induced neuroinflammation and pharmacological interventions such as cilostazol.
6. The use of different genders: There may be potential sex differences in the neuroinflammatory response and the efficacy of cilostazol's neuroprotective actions. The current study utilized only male rats, while including female animals could provide insights into potential sex-specific effects or vulnerabilities.
7. While the ELISA data implicate the p-Akt/p-GSK3*β*/p-CREB pathway, relying solely on this approach provides limited evidence of this specific mechanism. Future studies using complementary techniques such as Western blotting, pathway inhibitors/activators, and assessing downstream transcriptional effects could more definitively establish the role of this pathway.

By addressing these limitations in future research, extending to multiple animal models, species, sex, dosing, treatment durations, and deeper mechanistic studies, the findings could be further validated. This would ultimately strengthen the evidence supporting the clinical development of cilostazol for treating neuroinflammatory and neurodegenerative disorders. Overall, this work lays an important foundation demonstrating cilostazol's neuroprotective capabilities. Follow-up studies are logical next steps to provide a more complete evidence regarding cilostazol's therapeutic potential for debilitating neurological conditions.

## 5. Conclusion

In light of the present study, the cilostazol mitigated the neuropathological and behavioral disturbances induced by LPS in rats via curbing the AKT/GSK3*β*/CREB-induced neuroinflammation. Further mechanistic studies are warranted to emphasize the involvement of this signaling pathway.

## Data Availability

The data used to support the findings of this study are included within the article.
